# Wood’s Medical and Surgical Monographs

**Published:** 1890-07

**Authors:** 


**Wood's Medical and Surgical Monographs.** Vol. VI., No. 1, April, 1890. Vol. VI., No. 2, May, 1890. Vol. VI., No. 3, June, 1890. Published monthly. Price, $10.00 a year; single copies, $1.00. New York: William Wood & Co. 1890.

The first (April) number of volume six of these valuable periodicals contains: The Human Foot, its Form, Structure, Functions and Clothing, by Thomas R. Ellis, M. R. C. S.; Modern Cremation, its History and Practice, by Sir H. Thompson, F. R. C. S.; Aphasia: A Contribution to the Subject of the Dissolution of Speech from Cerebral Disease, by James Ross, M. D., LL. D. The first article is profusely illustrated with wood cuts and colored plates; it goes into the artistic anatomy of the foot with much detail, and is the most valuable contribution to this interesting subject that has yet appeared. The importance of properly caring for the foot is not properly appreciated by the majority of people, and from this essay can be gleaned the rarest and latest information on the subject. The other titles in this number are interesting and are very ably handled by their authors.

The second (May) number contains: Insanity at the Puerperal, Climacteric, and Lactational Periods, by William Bevan Lewis, L. R. C. P.; Treatment of Diseases of Women by Massage, by Dr. Robert Ziegenspeck, Munich; The Treatment of Internal Derangements of the Knee-Joint by Operation, by Herbert William Allingham, F. R. C. S.; The Idiopathic Enlargements of the Heart, by Dr. Oscar Fraentzel, Berlin. This number also contains illustrations, and discusses several interesting problems in the recent literature of medicine.

The third (June) number of volume six contains: Bronchial Asthma, its Causes, Pathology and Treatment, by John C. Thorowgood, M. D., F. R. C. P.; Convulsion Seizures, by J. Hughlings Jackson, M. D., F. R. C. P.; Surgical Treatment of Diseases of the Brain, by Ernest von Bergmann, Berlin. These are all interesting titles, and are discussed by men of eminence, whose opinions command respect. This number also contains the index for Volume VI.

